# Sex Determination in Japanese Quails (*Coturnix japonica*) Using Geometric Morphometrics of the Skull

**DOI:** 10.3390/ani12030302

**Published:** 2022-01-26

**Authors:** Tomasz Szara, Sokol Duro, Ozan Gündemir, İsmail Demircioğlu

**Affiliations:** 1 Department of Morphological Sciences, Institute of Veterinary Medicine, Warsaw University of Life Sciences-SGGW, 02-776 Warsaw, Poland; 2 Faculty of Veterinary Medicine, Agricultural University of Tirana, 1029 Tirana, Albania; durosokol@ubt.edu.al; 3 Department of Anatomy, Faculty of Veterinary Medicine, Istanbul University-Cerrahpasa, Istanbul 34500, Turkey; ozan.gundemir@iuc.edu.tr; 4 Department of Anatomy, Faculty of Veterinary Medicine, Harran University, Sanliurfa 63200, Turkey; idemircioglu@harran.edu.tr

**Keywords:** avian anatomy, bird, geometric morphometrics, principal component analysis, sex differentiation

## Abstract

**Simple Summary:**

This study aims to determine the sexual dimorphism of quail’s skull by using the geometric morphometric morphometrics and to reveal the shape differences between male and female individuals. For these purposes, photographs of quail skulls in dorsal, ventral, and caudal views were taken and analyzed with the geometric morphometric method. The most significant difference in terms of sexual dimorphism was observed in the dorsal aspect. In caudal view, it was seen that the roof of the skull in females is thinner and longer than in males, but the sex distribution was not decisive compared to other aspects. Geometric morphometrics proved to be a good tool for analyses of the sexual dimorphism and better than traditional morphometrics by their potential way of shape analyses.

**Abstract:**

The study investigated whether there is a morphological difference between the shape of the female and male quail’s skulls. For this purpose, 18 female and 21 male quails were used. After the skulls were obtained, their photographs were taken, and geometric analysis was performed. Dorsal (14 landmarks), caudal (8 landmarks), and ventral (13 landmarks) images of skulls were evaluated. As a result of the Principal Component Analysis (PCA), 28 principal components (PCs) were obtained for dorsal view, 16 PCs for caudal view, and 26 PCs for ventral view. PC1 (41.206%) for the caudal aspect explained the highest shape variation in terms of sex. It was seen that PC1 for dorsal view explained 33.046% and PC1 for ventral view explained 34.495% shape variation. For the dorsal view, the orbital pit of males was found to be deeper than females. The foramen magnum was narrower in female skulls. The lateral borders of the neurocranium were more pointed upwards in males. On ventral view, it was seen that male individuals had a wider skull in shape. Geometrically, it was determined that the male and female distinction was the best in the dorsal view. According to the dorsal view, only one male individual was found to be in the female group, and all other male individuals were completely separated from the females. After the dorsal view, the best distinction was seen ventrally. In the caudal examination, sexual discrimination was not fully seen. In this study, shape differences in quail’s skulls were examined between sexes, and shape differences were revealed geometrically. In addition to traditional morphometry studies, it is thought that geometric analysis studies will add a useful perspective to the literature.

## 1. Introduction

Japanese quail belongs to the order Galliformes, the family Phasianidae, and the genus *Coturnix*. Japanese quail were successfully introduced from Japan to America, Europe, and the Near and Middle East [1]. Domestication of Japanese quail began in Japan in the 11th century. While it was raised and cared for as a hobby in the first place, it has started to occupy an important place in the poultry industry with its short puberty age, low feed consumption, and high egg yield [2,3]. Because of these features, quails are also frequently used as experimental animals [4]. The Japanese quail (*Coturnix japonica* Temminck and Schlegel, 1848) is closely related to the common quail (*Coturnix coturnix* Linnaeus, 1758), a wild migratory bird inhabiting the “Old world” area. Until recently, they were classified as subspecies of one species (*Coturnix coturnix japonica* and *Coturnix coturnix coturnix*) [5]. They have now been recognized as two separates but closely related species of birds [6]. Both populations have allopatric distributions in wild. However, widespread breeding of Japanese quail in Europe, West Asia, and Northern Africa promotes the crossing of both wild and domesticated subspecies and the hybridization of genes, especially in the common quail population [7].

Unlike in mammals, the avian skeleton is adopted to active flight. This unique ability plays an important role in the conservation of avian species. Thanks to flying, birds gained access to food sources unattainable for terrestrial vertebrates. It also enables seasonal migrations of birds in search of optimal feeding areas. Due to such a specific method of locomotion, the birds developed a number of morphological features that distinguish them from other animals. Apart of the cortical bone and the cancellous bone, birds have a third type of bone tissue, called the medullary bone. The latter serves as a reservoir of calcium, which is particularly important in females in connection with the eggshell formation [8]. The quail’s skull, as in all bird skulls, consists of thin bony plates formed of either connective tissue or cartilaginous templates [9]. The shape of the head is aerodynamic, tapering rostrally. The facial bones are thin and usually very small but mainly depend on the shape and size of the bird’s beak. The bones of the neurocranium are completely fused with each other and the boundaries between the bones of the neurocranium are almost indistinguishable [10]. The avian skull consists of two parts, the neurocranium and the splanchnocranium, as in other vertebrates. The neurocranium in birds consist of os ccipital, os sphenoidale, os squamosum, os parietale, os frontale, paired ossa otica, unpaired os mesethmoidale, os ectethmoidale, and os lacrimale [11]. Orbits are very large, separated from each other by thin interorbital septum, and the braincase is relatively voluminous, especially in small birds. The cranium is connected to the vertebral column by a single occipital condyle, which gives a range of head movement that is unattainable by other vertebrates. The conformation of the facial skeleton is determined by the shape of the beak. A specific feature is the ability to raise the upper part of the beak due to the movable connection with the cranium. This is ensured by the maxillopalatine apparatus, the central part of which is the os quadratum. This apparatus is the equivalent of the mammalian temporomandibular joint [12]. Another flight adaptation is the presence of the air sacs found in some skeletal bones in poultry. In addition, these structures have a connection with the respiratory system [13].

Distinguishing the sex in quails like in other birds on the basis of the morphological features of the skull is quite difficult, if not practically impossible. We believe that the use of geometric morphometrics in skull images will be successful in differentiation of the sex in quails.

Craniometry is a commonly used tool in the taxonomic classification of vertebrates [14]. In this context, interspecific differences are defined on the base of morphometric data [15]. The skull is an object of both linear and geometric morphometrics in terms of interspecific comparisons and intraspecific polymorphism. Geometric morphometrics also allows the study of species patterns and evolutionary processes [16]. This method determines the amount of shape change by determining the shape and position differences of objects using landmark (LM) coordinates [17,18]. With General Procrustes Analysis (GPA), homologous landmarks of all samples are superimposed over a common center. This process removes the influence of factors such as direction, position, and size, allowing only shape differences to be revealed [19,20,21].

In recent years, geometric morphometric methods have been used frequently on various breeds and species to contribute to the taxonomic classification of animals and to determine the differences between sexes [21,22,23,24,25]. This study aims to determine the sex dimorphism of quail craniums by using geometric morphometrics method and to reveal the shape differences between male and female individuals.

## 2. Materials and Methods

### 2.1. Study Sample

In this study, 18 female and 21 male quails (*Coturnix japonica*) were used. The skulls of these birds were collected after slaughter from a private breeder’s farm in Albania. Maceration of the collected skulls was carried out at the Faculty of Veterinary Medicine, Agricultural University of Tirana. After the soft tissue and muscles were removed, the samples were prepared for photographing. Then, 2D pictures were captured perpendicular to the dorsal, caudal, and ventral aspects of the skull from a distance of 15 cm by the same device (Samsung photo camera NX210 20.3 MP) and by the same person. The images were then transferred to the electronic device for landmark operations.

### 2.2. Landmarks and Data Collections

**Figure 1:**
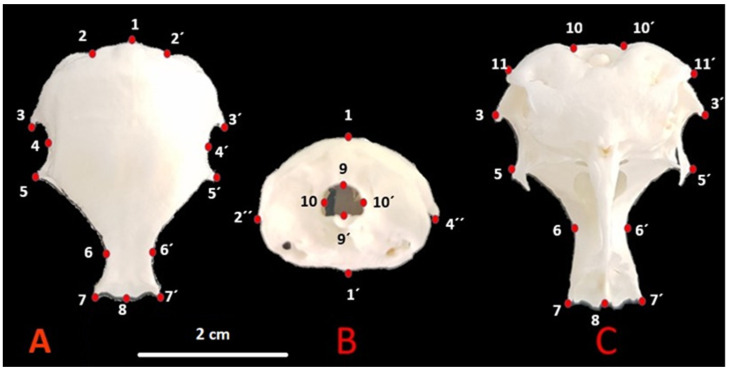
Skull landmarks in quail. (**A**) Dorsal, (**B**) caudal, (**C**) ventral.

The files were converted to tps format using the tpsUtil (version 1.74) program [26]. Then, the TpsDig2 software was used for landmark operations. A total of 14 landmarks (LM) were used for the dorsal view, 8 landmarks for the caudal view, and a total of 13 landmarks for the ventral view. The landmarks used according to *Nomina Anatomica Avium* [10] are shown in Figure 1.

(A). 1—Cerebellar prominentia; 2, 2′—Exoccipital bone; 3, 3′—Proc. suprameaticus of squamosal bone; 4, 4′—Temporal fossa; 5, 5′—Postorbital process; 6, 6′—Dorsal middle point of supraorbital margin; 7, 7′—Craniolateral terminal point of frontal bone; 8—Middle point of frontonasal suture;
(B). 1—Cerebellar prominentia; 1′—Basilar tuberculum of basioccipital bone; 2″, 4″—Postorbital process; 9, 9′—Foramen magnum high; 10, 10′—Foramen magnum width;
(C). 10, 10′—Foramen magnum width; 11, 11′—Paraoccipital process; 3, 3′—Procc. suprameaticus of squamosal bone; 5, 5′—Postorbital process; 6, 6′—Dorsal middle point of supraorbital margin; 7, 7′—Craniolateral terminal point of frontal bone; 8—Middle point of frontonasal suture.

### 2.3. Statistical Analysis

We performed Generalized Procrustes Analysis to remove differences in size, orientation, and positioning from our original landmark coordinates. Generate covariance matrix was applied. To visualize shape changes based on the position of the sex in the morphospace and to identify the major components of variation, a Principal Components Analysis (PCA) was performed. Shape and centroid size between the sexes were compared and were obtained with Procrustes ANOVA. Canonical variates analysis (CVA) was then used to reveal the differences between the sexes. Mahalanobis distances and Procrustes distances values and *p*-values were obtained from CVA. All analyzes were done with MorphoJ (v. 1.07a) [27].

## 3. Results

As a result of PCA analysis, 28 PCs were obtained for dorsal view, 16 PCs for caudal view, and 26 PCs for ventral view. PC1 for caudal view was 41.206%, which explained the highest shape variation in terms of sex. PC1 for dorsal view explained 33.046% and PC1 for ventral aspect explained 34.495% shape variation. The six PC eigenvalues describing the highest variations and their variances are given in Table 1.

**Figure 2:**
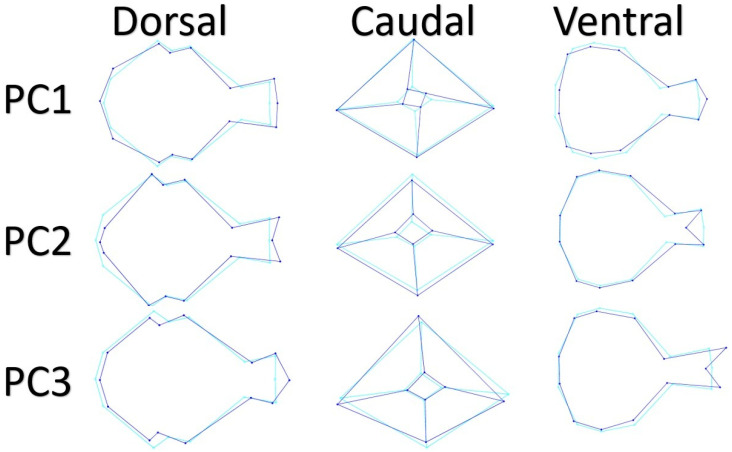
Wire-frame warp plots of changes in skull shape of PC1, PC2. And PC3. The darker blue plots indicate the positive limit of the principal component’s scores.

Wire-frame warp plots of changes in skull shape of PC1, PC2, and PC3 are shown in Figure 2. A high PC1 score for dorsal view represented a thinner and longer skull. Dorsal middle point of supraorbital margin (LM6) was lower for positive PC1. Points of the exoccipital bone (LM2) became wider as the PC1 value increased. The craniolateral terminal point of frontal bone (LM7) and middle point of the frontonasal suture (LM8) were more prominent as the PC1 value increased. In the PC2 high score, the middle point of the frontonasal suture (LM8) was more caudal than the craniolateral terminal point of the frontal bone (LM7). The PC2 positive score represented a more pointed neurocranium. In the PC3 high score, the middle point of frontonasal suture (LM8) was more cranial than the craniolateral terminal point of frontal bone (LM7).

A higher PC1 score for the caudal view represented a narrower foramen magnum. For PC1, the difference between the outer borders of the skull was not evident. In PC1 positive score, foramen magnum width (LM10) was more medial. In the high score for PC2, the distance between the cerebellar prominentia (LM1) and the foramen magnum was short. In addition, as the PC2 score increased, the point of cerebellar prominentia got closer to the ventral. Additional significant change for PC3 was in the postorbital process. As the PC3 score increased, the point of cerebellar prominentia got closer to the ventral like PC2.

A higher PC1 score for ventral view represented a narrower skull. There was also a more oval neurocranium with a high score for PC1. There was a marked difference for PC1 in the middle point of frontonasal suture. In positive PC1, the middle point of the frontonasal suture was more cranial. There were only three points difference for PC2 on the ventral view; the middle point of frontonasal suture and dorsal middle points of supraorbital margin. In PC2, the middle point of frontonasal suture was more caudal. Dorsal middle points of supraorbital margin were more inward and more caudal. The most important change for PC3 was at the craniolateral terminal point of the frontal bone. The positive score for PC3 was more cranial than the craniolateral terminal point of the frontal bone.

**Figure 3:**
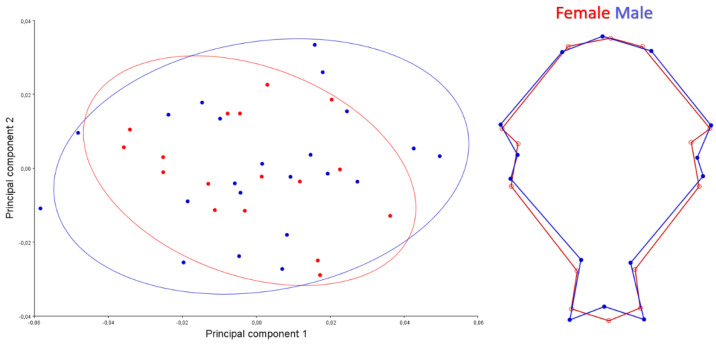
Scatter plot of PC1 (33.046) and PC2 (14.203) of quails for dorsal view (**left**). Wire-frame warp plots of changes in the cranial shape of quail, as mapped by 14 landmarks (**right**).

A scatter plot for the dorsal view is given in Figure 3. PC1-2 explained 47.25% of the total variation. Female skull samples showed wider shape change than male skulls. For the dorsal view, the orbital pit of males was found to be deeper than females.

**Figure 4:**
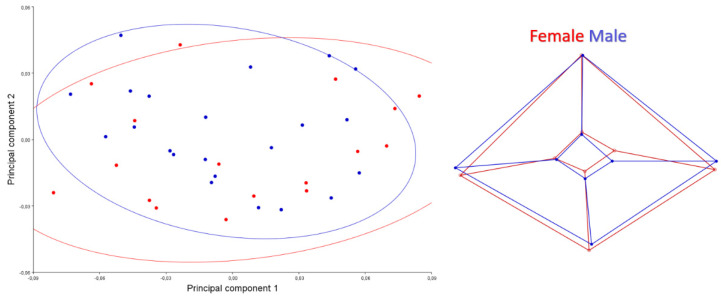
Scatter plot of PC1 (41.206) and PC2 (34.246) of quails for caudal view (**left**). Wire-frame warp plots of changes in the cranial shape of quail, as mapped by 8 landmarks (**right**).

A scatter plot for the caudal view is given in Figure 4. PC1-2 explained 75.45% of the total variation. Skulls of male samples showed wider shape change than females skulls samples. The foramen magnum was narrower in females’ skulls. The lateral walls of the neurocranium were more pointed upwards in males. It was seen that the roof of the skull of females was thinner and longer than in male individuals. This applies especially to the rostral part of the skull.

**Figure 5:**
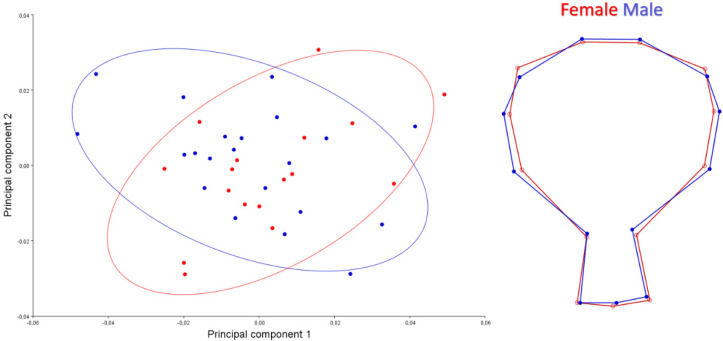
Scatter plot of PC1 (34.495) and PC2 (22.679) of quails for ventral view (**left**). Wire-frame warp plots of changes in the cranial shape of quail, as mapped by 13 landmarks (**right**).

A scatter plot for ventral is given in Figure 5. PC1-2 explained 57.17% of the total variation. Skulls of male samples showed wider shape change than females skulls samples. In ventral view, it was seen that male individuals had a bit wider skull in shape.

Procrustes ANOVA showed that skull size (as centroid size) differed significantly between the sexes in caudal view, whereas shape differed in dorsal view (Table 2). In other results, there was no difference in the size or shape of the skull between the sexes.

Centroid size and standard deviations results are given in Table 3. For analyzes performed in dorsal view, males had higher centroid size values than females. In the analyzes made in caudal and ventral views, the centroid size values of females were high.

**Figure 6:**
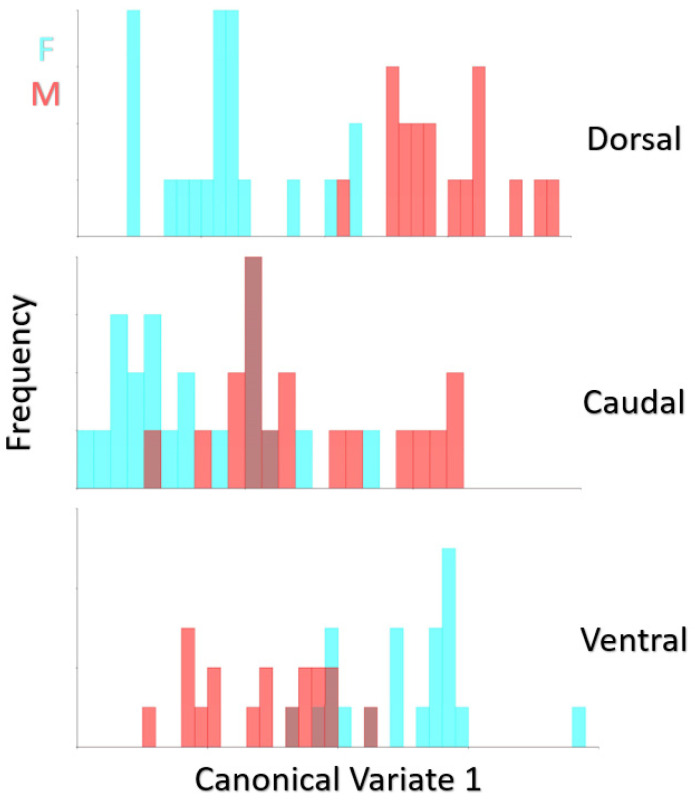
Frequency of distribution for canonical variate 1 of female quail (n:18) and male quail (n:21). (*p* < 0.0001 from 10,000 permutation rounds for Procrustes distances among groups).

Mahalanobis distances and Procrustes distances values and *p*-values are given in Table 4 (10,000 permutations). The difference between Procrustes distances was insignificant for sex. Mahalanobis distances were statistically significant in terms of dorsal and ventral aspects (*p* < 0.0001). The frequency of distribution for canonical variant 1 of female quail (n:18) and male quail (n:21) is given in Figure 6. In the Canonical variate results, it was seen that skulls of males and females differed better in the dorsal view. According to the dorsal view, only one male individual was found to be in the female group, and all other male individuals were completely separated from the females. After the dorsal view, the best distinction was seen ventrally. In the caudal examination, sexual discrimination was not fully seen.

## 4. Discussion

In this study, the quail skull was examined in terms of geometric morphometrics between the sexes. Shape variations between the two groups were examined, and the landmarks where the differences between the sexes were observed were examined in dorsal, caudal, and ventral views. In the study, it was observed that the shape variations of the male and female samples were close to each other. While the differentiation between males and females was more evident in the dorsal aspect, there was no definite differentiation in the caudal aspect. However, it can be said that geometric shape analysis is useful for sex discrimination within the same species. Using traditional morphometric methods in various skulls, the differences between males and females observed in longitudinal measurements [28,29,30,31]. In such studies, most of the dimensions in males were longer than females. However, thanks to geometric analysis, it could be interpreted in terms of shape. To determine the sexual dimorphism by geometric analysis in dogs, goats, and sheep, information was obtained in terms of geometric morphometry [21,32,33,34].

The bones of various species of birds have been the subject of detailed scientific analysis for many years [35]. In addition to the features common to all Aves, researchers are trying to find similarities and differences between sometimes distant systematic groups [36]. Rarely, however, the authors are able to indicate features of the skull or postcranial skeleton that allow differentiating males from females. In a study conducted in the turkey neurocranium, sexual dimorphism was tried to be determined using geometric analysis [37]. In our study performed on the quail, can be seen that male individuals have wider skulls in caudal view. Likewise, in the quail, occipital height in females was bigger than in males, similar as in turkeys. The roof of the skull was wider in males and higher in females. In the ventral view, as in the turkeys, the skulls of the males were wider in the quail. In a study performed on turkey skulls, it was reported that PC1 explained 39.61% of all variation in dorsal view, 51.9% in caudal view, and 34.32% in ventral view. In quail’s, these rates were 33.05%, 41.21%, and 34.5%, respectively. PC1 caudal appearance explained the highest shape variation in both studies. In these two studies, the most significant gender differentiation in caudal appearance was in the foramen magnum. Sexual dimorphism of the foramen magnum was mentioned in a morphometric study in humans [38]. On the other hand, whether the foramen magnum shows sexual dimorphism in poultry may be the subject of further geometric morphometry and morphometric studies.

Lau et al. [39] made similar observations in otters. However, they noticed that size dimorphism is greater than shape dimorphism. The size differences in the skull of males and females are quite common in animals [15,31]. Livezey and Humphrey [40] noticed that the Continental Streamer-ducks’ sexual dimorphism was mainly related to body size. Males are larger but have proportionately smaller skulls. Similar conclusions were presented by Johnston [41]. While examining Rock Doves and feral pigeons, he observed size dimorphism of varying severity. Shape dimorphism was poorly marked, with female skulls being shorter and narrower than males. In quails, unlike other Galliformes, females are larger than males [42]. The differences, however, are not significant and intraspecific variability, age influence, as well as selection for the bodyweight [43] or hybridization process, make sexual differentiation in Japanese quail based on body size almost impossible. Therefore, our proposal for determining the sexual shape dimorphism using geometric morphometry seems all the more useful.

## 5. Conclusions

In this study, we tried to reveal sexual dimorphism by using the geometric morphometry method on quail skulls. Shape variations between the sexes were obtained from the skulls examined in three different views. Significant differences in sex distribution were observed in the dorsal view. Studies of geometric morphometry will be useful in taxonomy or sexual dimorphism studies and will bring a different perspective to traditional morphometric studies.

## Figures and Tables

**Table 1:** Principal Components (PC) Eigenvalues describing the highest variation (V) for dorsal, caudal, and ventral view, as a result of principal component analysis, and variation values it explains.

PC	Dorsal		Caudal		Ventral	
	Eigenvalue	% V	Eigenvalue	% V	Eigenvalue	% V
1	0.000532077	33.046	0.00235293	41.206	0.000484881	34.495
2	0.000228691	14.203	0.00195548	34.246	0.000318786	22.679
3	0.000208817	12.969	0.000515875	9.344	0.000128329	9.1295
4	0.000125772	7.8113	0.000364257	6.3791	0.000106107	7.5486
5	0.000105952	6.5804	0.000159492	2.7931	7.22557 × 10^−5^	5.1404
6	8.52045E-05	5.2918	0.000106751	1.8695	5.58247 × 10^−5^	3.9714

**Table 2:** Procrustes ANOVA results (Bolded: significant difference).

Individuals	Database		F	*p*-Value
Sex	Dorsal	Centroid Size	0.01	0.9136
		Shape	1.83	**0.0092**
	Caudal	Centroid Size	4.87	**0.0336**
		Shape	0.56	0.8717
	Ventral	Centroid Size	2.07	0.1584
		Shape	0.79	0.7406

**Table 3:** Centroid size and standard deviations of quails.

		Dorsal	Caudal	Ventral
**Female**	Mean	14,975.44	8230.163	16,311.38
	SD	6112.519	2656.018	6623.777
**Male**	Mean	17,561.24	7113.946	15,894.75
	SD	526.8477	3880	6784.003

**Table 4:** Mahalanobis distances and procrustes distances values and *p*-values.

	MD	MD-P	PD	PD-P
Dorsal	3.5694	<0.0001	0.0178	0.0510
Caudal	1.3809	0.0751	0.0167	0.4923
Ventral	2.2510	<0.0001	0.0100	0.5791

MD: Mahalanobis distances among the group, MD-P: *p*-values from permutation tests (10,000 permutation rounds) for Mahalanobis distances among group, PD: Procrustes distances among group, PD-P: *p*-values from permutation tests (10,000 permutation rounds) for Procrustes distances among group.

## Data Availability

The data presented in this study are available on request from the corresponding author.
